# Genomic Organization of Microsatellites and *LINE-1-*like Retrotransposons: Evolutionary Implications for *Ctenomys minutus* (Rodentia: Ctenomyidae) Cytotypes

**DOI:** 10.3390/ani12162091

**Published:** 2022-08-16

**Authors:** Thays Duarte de Oliveira, Natasha Avila Bertocchi, Rafael Kretschmer, Edivaldo H. C. de Oliveira, Marcelo de Bello Cioffi, Thomas Liehr, Thales R. O. de Freitas

**Affiliations:** 1 Programa de Pós-Graduação em Biologia Animal, Universidade Federal do Rio Grande do Sul, Porto Alegre 91501-970, Brazil; 2 Programa de Pós-Graduação em Genética e Biologia Molecular, Universidade Federal do Rio Grande do Sul, Porto Alegre 91501-970, Brazil; 3 Departamento de Ecologia, Zoologia e Genética, Instituto de Biologia, Universidade Federal de Pelotas, Pelotas 96010-900, Brazil; 4 Instituto de Ciências Exatas e Naturais, Universidade Federal do Pará, Belém 66075-110, Brazil; 5 Laboratório de Cultura de Tecidos e Citogenética, SAMAM, Instituto Evandro Chagas, Ananindeua 67030-000, Brazil; 6 Departamento de Genética e Evolução, Universidade Federal de São Carlos, São Carlos 13565-905, Brazil; 7 Institute of Human Genetics, University Hospital Jena, Friedrich Schiller University, 07747 Jena, Germany

**Keywords:** chromosomal rearrangements, FISH, *LINE-1*, simple sequence repeats, retrotransposons

## Abstract

**Simple Summary:**

In animals, several species contain substantial chromosomal and genomic variation among their populations, but as to what could have driven such diversification is still a puzzle for most cases. Here, we used molecular cytogenetic analysis to expose the main genomic elements involved in the population variation observed in the Neotropical underground rodents of the genus Ctenomys (Rodentia: Ctenomyidae), which harbor the most significant chromosomal variation among mammals (2n = 10 to 2n = 70). These data provide evidence for a correlation between repetitive genomic content and localization of evolutionary breakpoint regions (EBRs) and highlight their direct impact in promoting chromosomal rearrangements.

**Abstract:**

The Neotropical underground rodents of the genus *Ctenomys* (Rodentia: Ctenomyidae) comprise about 65 species, which harbor the most significant chromosomal variation among mammals (2n = 10 to 2n = 70). Among them, *C. minutus* stands out with 45 different cytotypes already identified, among which, seven parental ones, named A to G, are parapatrically distributed in the coastal plains of Southern Brazil. Looking for possible causes that led to such extensive karyotype diversification, we performed chromosomal mapping of different repetitive DNAs, including microsatellites and long interspersed element-1 (*LINE-1*) retrotransposons in the seven parental cytotypes. Although microsatellites were found mainly in the centromeric and telomeric regions of the chromosomes, different patterns occur for each cytotype, thus revealing specific features. Likewise, the *LINE-1*-like retrotransposons also showed a differential distribution for each cytotype, which may be linked to stochastic loss of *LINE-1* in some populations. Here, microsatellite motifs (A)_30_, (C)_30_, (CA)_15_, (CAC)_10_, (CAG)_10_, (CGG)_10_, (GA)_15_, and (GAG)_10_ could be mapped to fusion of chromosomes 20/17, fission and inversion in the short arm of chromosome 2, fusion of chromosomes 23/19, and different combinations of centric and tandem fusions of chromosomes 22/24/16. These data provide evidence for a correlation between repetitive genomic content and localization of evolutionary breakpoints and highlight their direct impact in promoting chromosomal rearrangements.

## 1. Introduction

Repetitive DNAs consist of identical or similar sequences arranged in tandem or dispersed throughout genomes, including transposable elements (TEs), multigene families, satellites, minisatellites, and microsatellites [1], representing 50% of the *Homo sapiens* [2] and 40% of the *Rattus norvegicus* genomes [3,4]. Even though it has been considered as “genomic junk” for a long time, it is now known that the repetitive fraction of the genome is composed of both functional and non-coding sequences. Its functionality is associated with gene expression regulation, recombination, sex chromosome differentiation, genomic instability, and chromosomal evolution [5,6,7,8,9].

Repetitive sequences can be distributed throughout the genome, but they are ubiquitously present in the heterochromatin [10], with a preferential location in the centromeric and pericentromeric regions in a wide variety of vertebrate groups such as fishes, reptiles, birds, and mammals including rodents [9,11,12,13]. However, TEs can be distributed in both euchromatic and heterochromatic regions of the chromosomes, depending on the characteristics of each TE group and independent of the host genome [14,15]. 

Retrotransposons can mobilize via RNA, and they are the most abundant TEs in vertebrate genomes, probably due to their “copy and paste” replication mechanism, resulting in new copies with each replication [16]. In mammals, the long interspersed element-1 (*LINE-1*) is the most abundant transposable element in the order of LINEs (Long INterspersed Elements) [17,18], and is potentially autonomous, since it has the coding for the enzymes needed for replication [16]. Although found in vertebrate genomes, *LINE-1* had a proliferative success in mammals, and is the only active LINE retrotransposon in humans [18,19]. *LINE-1* is believed to have harmful, neutral, and beneficial effects, as it is linked to heterochromatin formation, pseudogenes, chromosomal rearrangements, and human diseases [14,20,21,22,23]. As a result, *LINE-1* is generally associated with genome plasticity and chromosomal alterations [24].

Animal genomes present a great diversity in chromosomal number, size, and morphology. However, the diploid number (2n) is generally constant within a species and often variable among species [25]. Notably, the subterranean rodents of the Neotropical genus *Ctenomys* (Rodentia: Ctenomyidae) represent an exception to this general rule. This genus comprises approximately 65 species, showing the greatest chromosomal variation among mammals [26]. In fact, the 2n values varies from 10 in *C. steinbachi* to 70 in *C. pearsoni* [27,28]. In addition, variations are also found within the same species as, for example, in *C. minutus* (2n = 42 to 50), *C. talarum* (2n = 44 to 48), and *C. lami* (2n = 54 to 58) [29,30,31,32,33].

**Figure 1:**
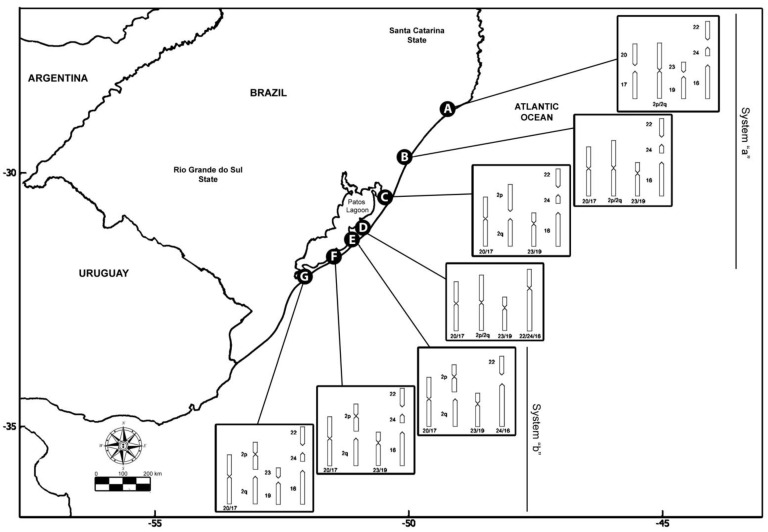
Geographic origin and karyotype rearrangements of the specimens of *Ctenomys minutus* analyzed in this study. Map of the coastal plain of Southern Brazil was obtained from https://earthobservatory.nasa.gov/map#6/−31.559/−48.011 (accessed on 12 June 2020), according to the NASA Image Use Policy (https://earthobservatory.nasa.gov/image-use-policy (accessed on 12 June 2020)).

The species *C. minutus* is restricted to Southern Brazil, inhabiting the sandy fields and dunes of the coastal plains of the states of Santa Catarina and Rio Grande do Sul [34]. It presents a notable chromosomal variation, with 45 different karyotypes (cytotypes) already described, the largest variation detected for the genus so far [31,35,36,37]. Seven parapatric parental karyotypes, here designated as cytotypes A to G, have been identified [31,37,38], with six hybrid zones also documented [29,31,36,37,38,39,40]. The progressive decrease of 2n from 50 to 42 chromosomes involved Robertsonian rearrangements, tandem fusions/fissions, and paracentric and pericentric inversions (Table 1 and Figure 1) [29,31,38,39], which were characterized by G-bands highlighting the chromosomal homologies among cytotypes [29,31,37]. Each cytotype is found in a particular geographic area, and the divergent populations can be separated by geographic barriers or have a contiguous distribution [29]. 

What could have driven the extensive karyotype diversification observed in these populations? Although the mapping of repetitive sequences has been shown to be useful for detecting karyotypic changes during the chromosomal evolution [41,42], such analyses are still scarce among rodents. As an example, the main 96 repetitive DNA family responsible for rolling circle replication in *Ctenomys* was isolated and characterized [43,44]. The aim was to investigate the distribution of different repetitive DNAs in order to characterize evolutionary breakpoint regions (EBRs) and to highlight their involvement in promoting chromosomal rearrangements among the seven parapatric parental cytotypes A–G. Additionally, chromosomal mapping of eight microsatellite sequence motifs and *LINE-1*-like retroelement was documented among the populations throughout their distribution area (coastal plains of Southern Brazil) here for the first time. Our results demonstrated that DNA satellites are tightly associated with mapped-in EBRs and most likely fostered the extensive karyotype diversification observed. 

## 2. Materials and Methods

### 2.1. Sample Collection

Thirteen individuals of *C. minutus* were collected in the states of Santa Catarina (SC) and Rio Grande do Sul (RS) (Table 2), using Oneida Victor^®^ n°0 weft traps. Animals were euthanized following the guidelines of the Animal Care Committee of the American Society of Mammalogists [45]. The experiments were conducted with the approval of the Ethics Committee for the Use of Animals (CEUA) n° 35,828 of the Universidade Federal do Rio Grande do Sul (Porto Alegre-Brazil), and all field procedures had appropriate permissions from Brazil’s Environmental Agency (IBAMA, Authorization n° 14690-1).

### 2.2. Chromosomal Preparations 

Chromosomal preparations were obtained from short-term fibroblast cultures [46], with modifications. Tissues from the kidney and/or the lung were disaggregated in collagenase type IV, cultured at 37 °C in DMEM—Dulbecco’s Modified Eagle’s Medium—high glucose (GIBCO™, Grand Island, NY, USA), enriched with 20% fetal bovine serum (GIBCO™, BRL), penicillin (100 units/mL), and streptomycin (100 mg/mL). Cells were grown to ~80–90% confluent. For further passages, cells were removed by adding Trypsin–EDTA (GIBCO™, USA), and up to five passages were performed. And at each passage, the chromosomes were obtained by standard protocols: cells were incubated for 3 h with colchicine, treated for 8 min in a hypotonic solution (0.075 M KCl), and fixed in a methanol and acetic acid (3:1) solution. To confirm the diploid number and check if there were no chromosomal alterations, approximately 30 metaphases of each individual were analyzed using 5% Giemsa staining in 0.07 M phosphate buffer (pH 6.8).

### 2.3. Probe Preparation and Fluorescence In Situ Hybridization (FISH)

The oligonucleotide probes containing the microsatellite motifs (A)_30_, (C)_30_, (CA)_15_, (CAC)_10_, (CAG)_10_, (CGG)_10_, (GA)_15_, and (GAG)_10_ were directly labeled with Cy3 during synthesis (Sigma-Aldrich, St. Louis, MO, USA) and hybridized to *C. minutus* metaphases, according to Kubat et al. [47]. After denaturation, the probes were applied to the slides and incubated for 16 h at 37 °C in a humid chamber. The slides were washed twice in 2× SSC, twice in 1× SSC and in PBS (phosphate-buffered saline), and then dehydrated in an ascending ethanol series (70, 90, and 100%) at room temperature. The chromosomes were counterstained by Fluoroshield™ with DAPI (Sigma–Aldrich).

The *LINE-1*-like retroelement was identified and amplified by the polymerase chain reaction (PCR) when using *C. minutus* (Cytotype A—50a) genomic DNA, which was isolated following [48] and with the set of primers as described by Casavant et al. [49]. The amplified product represented part of the TE reverse transcriptase. The amplicons of approximately 740 bp were purified with a GE Healthcare illustra™ GFX PCR DNA and Gel Band Purification Kit (GE Healthcare UK Ltd., Buckinghamshire, UK), following the manufacturer’s recommendations, and then sent for sequencing at Macrogen Inc. (Seoul, Korea). BLAST searches were conducted using the sequencing product against GenBank (https://www.ncbi.nlm.nih.gov/ (accessed on 8 June 2020)) to confirm the identity of the element. Additionally, ORF Finder (http://www.ncbi.nlm.nih.gov/gorf/gorf.html (accessed on 8 June 2020)) tools were utilized to verify the TE and the domains were identified using the Conserved Domain Database (CDD) platform https://www.ncbi.nlm.nih.gov/Structure/cdd/wrpsb.cgi (accessed on 8 June 2020). The *LINE-1*-like retrotransposons (have been deposited in GenBank—OP068276) were used as a template for the PCR labeling; the probe for FISH probe was labeled directly by PCR using Biotin-16-dUTP (Jena Bioscience, Jena, Germany).

The slide preparations, *LINE-1-*like hybridization, and post-hybridization were performed according to Bertocchi et al. [15], with minor modifications. The hybridization was performed overnight at 37 °C in a humid chamber, and the post-hybridization washes were carried out at 37 °C in 50% formamide for 3 min, followed by two washes in 2× SSC for 5 min each, at 37 °C. The signal was detected using streptavidin-Cy3, and the chromosomes were counterstained by Fluoroshield™ with DAPI (Sigma–Aldrich). For both microsatellites and *LINE-1*-like FISH experiments, at least 30 metaphases per individual were analyzed to confirm the FISH results and we built karyotypes for each cytotype to ensure correct identification of each chromosome. The slides were analyzed using a Zeiss Axiophot epifluorescence microscope (Zeiss Inc. Carl Zeiss, Heidelberg, Germany), coupled with ZEN BLUE software. Figures were organized using Adobe Photoshop CS6.

## 3. Results

**Figure 2:**
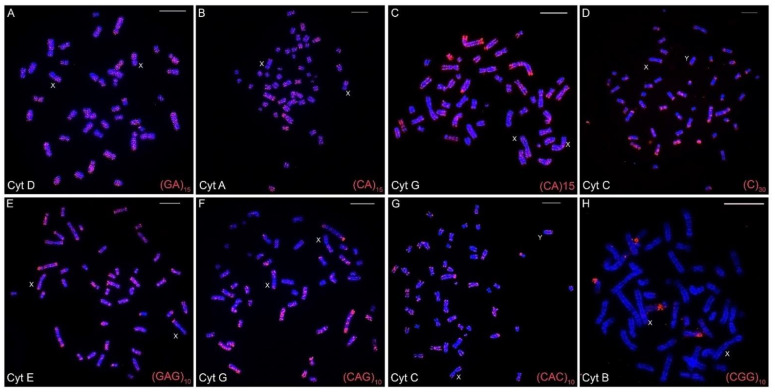
Fluorescence in situ hybridizations with varied microsatellites motifs in the different cytotypes (**A**–**H**) of *Ctenomys minutus*. The sex chromosomes are indicated for each metaphase. Bar = 10 μm.

**Figure 3:**
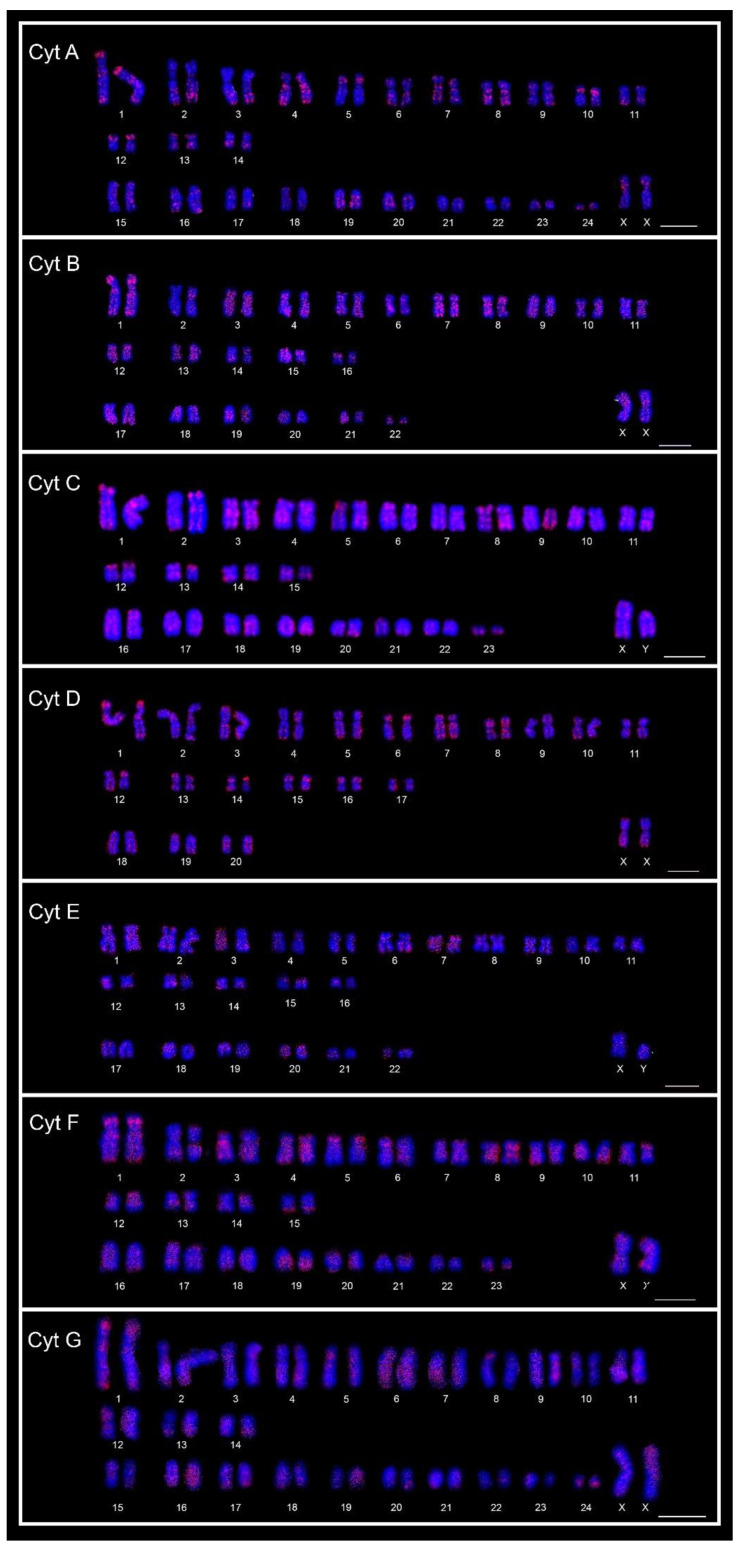
FISH karyotypes of the seven cytotypes (Cyt A–G) of *Ctenomys minutus* with the A_30_ motif probe. Bar = 10 μm.

**Figure 4:**
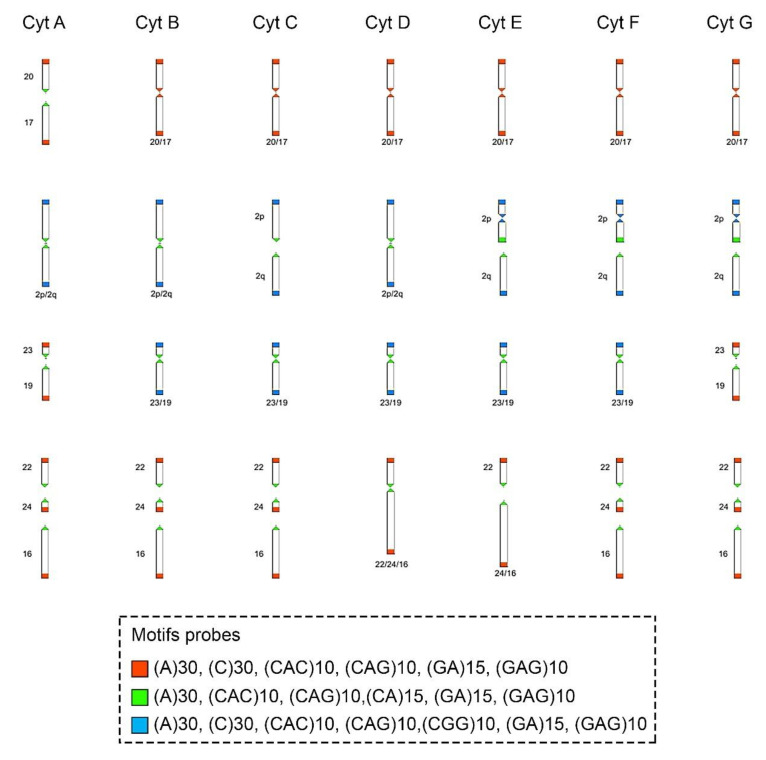
Schematic representation of the hybridization pattern of the microsatellite motif probes on the chromosomes of *Ctenomys minutus*. Comparison of the chromosomal rearrangements found among the cytotypes (Cyt A–G). The cytotype A was used as a standard to compare the chromosomal rearrangements in the other cytotypes. Modified from Freitas, Freygang et al., and Lopes et al. [29,31,37]. In the fusion 20/17 (line 1), the motifs involved are (A)_30_, (C)_30_, (CAC)_10_, (CAG)_10_, (GA)_15_, and (GAG)_10_ (blocks in red). In the fission of chromosome 2 (line 2), the motifs involved are (A)_30_, (CAC)_10_, (CAG)_10_, (CA)_15_, (GA)_15_, and (GAG)_10_ (blocks in green) and in the inversion in the short arm of chromosome 2 (line 2), the motifs involved are (A)_30_, (C)_30_, (CAC)_10_, (CAG)_10_, (CGG)_10_, (GA)_15_, and (GAG)_10_ (blocks in blue). In the fusion 23/19 (line 3), the motifs involved are (A)_30_, (CAC)_10_, (CAG)_10_, (CA)_15_, (GA)_15_, and (GAG)_10_ (blocks in green). In different combinations of centric and tandem fusions 22/24/16, the motifs involved are (A)_30_, (CAC)_10_, (CAG)_10_, (CA)_15_, (GA)_15_, and (GAG)_10_ (blocks in green), and (A)_30_, (C)_30_, (CAC)_10_, (CAG)_10_, (GA)_15_, and (GAG)_10_ (blocks in red).

The chromosomal mapping of the eight distinct microsatellite motifs revealed that distinct cytotype-specific patterns can occur, although they are generally accumulated in the centromeric and telomeric regions of the chromosomes (Table 3, Figure 2A–H, Figure 3, Figure 4 and Figures S1–S7). Only for the (CGG)_10_ probe, no signals of hybridization were observed in the sex chromosomes in any of the seven cytotypes; for the other seven microsatellite probes, signals were observed in the centromeric and terminal regions of the sex chromosomes in the seven cytotypes. 

The (CA)_15_ microsatellite shows hybridization signals spread over the entire length of the chromosomes, mainly in the largest ones (Figure 2B,C). However, the signals varied according to cytotypes. Cytotypes from the north of the geographic distribution present a different distribution when compared to cytotypes from the southern distribution. This difference can be seen, for example, when the distribution of (CA)_15_ in the cytotype A (50a) is compared with the distribution in the cytotype G (50b). Cytotype A visibly presents more and larger (CA)_15_ blocks, in contrast to cytotype G (Table 3, Figure 2B and Figure 2C, respectively, and Figures S1–S7). 

On the other hand, the microsatellites (C)_30_, (GAG)_10_, (CAG)_10_, and (CAC)_10_ show a spread distribution, with a preferential accumulation in the telomeric, centromeric, and pericentromeric regions of almost all of the chromosomes, with no significative differences among cytotypes (Table 3, Figure 2 and Figures S1–S7). Otherwise, the (CGG)_10_ microsatellite motif was found on chromosome pair 8 with secondary constrictions (i.e., nucleolus organizer region (NOR) carriers) in all cytotypes, and in a few additional chromosomal pairs other than in cytotype F. Additionally, (CGG)_10_ represents the only motif that did not accumulate in the sex chromosomes (Table 3, Figure 2). 

The motifs (GA)_15_ and (A)_30_ (Figure 2A, Figure 3 and Figure 4, respectively) are in the telomeric, centromeric, and pericentromeric regions of most of the chromosomes, with more extensive blocks in the largest chromosome pairs of karyotypes, in all cytotypes (Table 3 and Figures S1–S7). No cytotype-specific signals appear to occur for these motifs. In summary, at least six microsatellite motifs are accumulated in near all chromosomes involved in rearrangements previously described and directly involved in the origin of the different cytotypes, as shown in Figure 4. In the fusion points of chromosomes 20/17, we showed the motifs (A)_30_, (C)_30_, (CAC)_10_, (CAG)_10_, (GA)_15_, and (GAG)_10_ (Figure 4, line 1—blocks in red). In the fission of chromosome 2, the motifs involved are (A)_30_, (CAC)_10_, (CAG)_10_, (CA)_15_, (GA)_15_, and (GAG)_10_ (Figure 4, line 2—blocks in green) and in the inversion in the short arm of chromosome 2, the motifs involved are (A)_30_, (C)_30_, (CAC)_10_, (CAG)_10_, (CGG)_10_, (GA)_15_, and (GAG)_10_ (Figure 4, line 2—blocks in blue). In the fusion of chromosomes 23/19, the motifs involved are (A)_30_, (CAC)_10_, (CAG)_10_, (CA)_15_, (GA)_15_, and (GAG)_10_ (Figure 4, line 3—blocks in green). Furthermore, in different combinations of centric and tandem fusions of chromosomes 22/24/16, the motifs observed are (A)_30_, (CAC)_10_, (CAG)_10_, (CA)_15_, (GA)_15_, and (GAG)_10_ (Figure 4—blocks in green), and (A)_30_, (C)_30_, (CAC)_10_, (CAG)_10_, (GA)_15_, and (GAG)_10_ (Figure 4—blocks in red).

**Figure 5:**
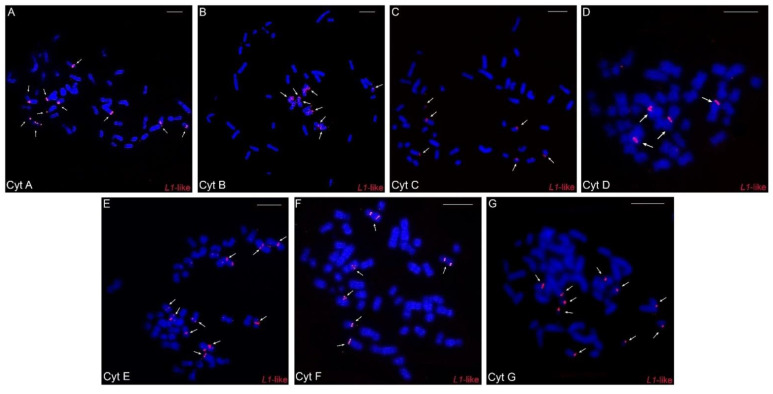
Fluorescence in situ hybridization experiments with the *LINE-1-*like probes in the different cytotypes (**A**–**G**) of *Ctenomys minutus*. The arrows indicate the *LINE-1*-like hybridization signals. Bar = 10 μm.

Concerning *LINE-1*-like repeats, in general, few hybridization signals were observed, with a preferential location in one of the distal chromosomal regions: in five pairs in cytotypes A, F, and G, in four pairs in cytotype B, in three pairs in cytotypes C and E, and in only two pairs in cytotype D (Figure 5A–G). 

## 4. Discussion

In mammals, chromosomal structural changes are usually related to repetitive and mobile sequences [50], especially in fragile sites [51], which consist of tandem sequences and TEs that can induce chromosomal breaks [52,53]. Indeed, in humans, primates, and mice, several EBRs have been found, evidencing that genomic reorganizations occur mainly in such regions where duplications/expansion of repetitive sequences occur [52,54,55,56,57]. Despite the importance of repetitive sequences in chromosomal evolution, the extensive karyotype variability found in *C. minutus* is still poorly investigated under a molecular cytogenetic approach.

Our current data show that at least six microsatellite motifs are altered in the chromosomes of *C. minutus* (Figure 4), principally in centromeric and telomeric regions, whose rearrangements were previously described [31]. The creation of loops and/or the mispairing of tandem repeats, which result in disruption-induced replication instability disorders primarily at telomeres, centromeres, and microsatellites, have been demonstrated to be associated with some neurological disorders in humans [58]. In addition, there are several examples and causes of instability of tandem repeats, owing to their intrinsic composition of sequences, secondary DNA structures, topological and physical aspects of tandem repeats, and other characteristics that can influence cellular processes and repair pathways, promoting rapid mutagenesis of the tandem repeats (see review in [59]). It has been shown that tandem repeats can mainly affect the replication mechanism during DNA synthesis, causing instability in the genome [60]. Some genomic sequences/motifs temporarily slow or stop the replication fork, increasing the likelihood of a double-strand break [61].

It is known that common fragile sites are enriched with A/T sequences and they can form secondary structures that enable chromosomal instability [52,62,63]. The A/T pairing tends to be more fragile, not only because of the number of connections, but also because of the stacking of bases [64]. Although specific repetitive sequences have not yet been seen as uniquely responsible for karyotype instability, our results showed that different microsatellite DNA motifs are found at the breakpoints in *C. minutus*, as observed with the monomer (A)_30,_ which is localized in all breakpoints of all cytotypes (Figure 2A–G, Figure 3 and Figure 4). 

The distribution of the *LINE-1-*like retrotransposons does not have a preferential location in heterochromatic regions, as seen with other retrotransposons such as *Rex1*, *Rex3*, and *Rex6* in fish genomes ([65], reviewed in [66]), *CR1* in the woodpecker genome [15], and SINE-B1 in rodents of the genus *Proechimys* [67]. The dispersed chromosomal distribution of *LINE-1* may be a common characteristic for rodents, since this distribution has also been observed in other species such as *Tatera gambiana* (Muridae, Gerbillinae), *Acomys* sp. (Muridae, Deomyinae), *Cricetomys* sp. (Nesomyidae, Cricetomyinae), *Microtus arvalis* (Cricetidae, Arvicolinae), *Phodopus roborovskii*, and *P. sungorus* (Cricetidae, Cricetinae) [68]. The presence of *LINE-1* in both eu- and heterochromatic chromosomal regions may be due to its involvement with the repetitive tandem DNA (satellites, microsatellites, and minisatellites). Furthermore, its distribution in the euchromatin may indicate the occurrence of active *LINE-1* copies [50,69]. 

A differential distribution of the *LINE-1*-like element was observed for each cytotype. It is more accumulated in cytotypes A, F, and G (larger 2n) than in others (Figure 5A–G), with cytotype D (smallest 2n) having the lowest number of signals. (Figure 5A–G). Thus, we observed that there is an association between the number of *LINE-1*-like signals and 2n, an association similar to the one that occurs between 2n and the geographic distribution of the species, where the extremities have higher numbers and there is a progressive decrease along the distribution and then a further progressive increase (Figure 5). Here, we suggest that such an uneven distribution may be linked to a vertical transmission of *LINE-1*-like signals to all populations of *C. minutus*, since the presence of this retroelement precedes the divergence of marsupials and eutherians [17]. Considering that the *LINE-1* element was present in a *Ctenomys* ancestor, it is likely that it has mutated, progressively producing fewer active copies. As a result, the occurrence of retroelements is decreasing in the populations and, probably, undergoing an extinction process in *Ctenomys.*

Our results also indicate that the number of *LINE-1*-like elements has an association with the diploid number of cytotypes (Figure 5A–G). Our results do not suggest that *L1* hybridization regions on the chromosomes are breakpoints, but rather that the lack of *L1* may have provided the ideal environment for such breaks. Considering the variation in the number of signals between the different cytotypes seen in our FISH experiments (Figure 5A–G) and that *LINE-1* plays an important role in the DNA repair [66], we raised the hypothesis: considering that the cytotype A is more similar to the ancestor of *C. minutus* [29], the *LINE-1* sequences were possibly lost or are very degenerated in the other cytotypes, thus favoring chromosomal rearrangements and the emergence of other cytotypes. Previous studies have also reported that the loss or inactivation of *LINE-1* increased the chromosomal diversity, as found in the rodents of the Muroid group [70], and *Oryzomys* and *Holochilus* [49]. In addition, species with large numbers of sequenced genomes, e.g., humans from different geographic origins, showed drastic variation in the activity of some *L1*-like elements, contributing to human genetic variability [71]; all these observations corroborate our hypothesis. 

## 5. Conclusions

This study provided evidence for a direct spatial correlation between the repetitive DNA and EBRs, highlighting their direct impact in promoting chromosomal rearrangements and the divergence of cytotypes of *C.* *minutus*. Recently, similar observations were made for songbirds of genus *Senna* [72]. However, although the first step towards understanding the coevolution between *LINE-1* and the host genome has also been taken, it is still necessary to deepen the knowledge about the structural characteristics of *LINE-1* copies, such as Southern blot, ORF isolation, and the estimation of the age of the youngest elements, to better understand this question within the *Ctenomys* model.

## Figures and Tables

**Table 1:** Karyotype data with respective cytotype, diploid number (2n), karyotype, Fundamental Number (NF), and the main rearrangements involved in their differentiation.

Cytotype	2n	Karyotype	NF	Fusion	Fission
Cytotype A	50 a	28 sm + 20 a; XY	76		Standard
Cytotype B	46 a	32 sm + 12 a; XY	76	20/17; 23/19	-
Cytotype C	48 a	30 sm + 16 a; XY	76	20/17; 23/19	2p; 2q
Cytotype D	42	36 sm + 6 a; XY	78	20/17; 23/19; 22/24/16	-
Cytotype E	46 b	32 sm + 12 a; XY	76	20/17; 23/19; 24/16	2p *; 2q
Cytotype F	48 b	30 sm + 16 a; XY	76	20/17; 23/19	2p *; 2q
Cytotype G	50 b	28 sm + 20 a; XY	76	20/17	2p *; 2q

Cytotypes with the same 2n are described with “a” or “b”, due to the rearrangements in the different chromosomes, allowing them to be differentiated. System “b” chromosome 2 is fissioned, giving rise to two chromosomes (corresponding to 2p and 2q) and a pericentromeric inversion in the chromosome corresponds to 2p. sm = submetacentric; a = acrocentric; * denotes inversion. The karyotype data and the rearrangements involved in their differentiation are by Freitas, Freygang et al., and Lopes et al. [29,31,37].

**Table 2:** Collection sites for the analyzed *C. minutus* cytotypes, together with the respective sample sizes.

Cytotype	Individuals/Sex	Locality	Geographic Coordinate
A	1 ♀	Jaguaruna (SC), Brazil	28°41′53.02″ S 49°01′33.86″ W
B	2 ♀	Praia do Barro (RS), Brazil	29°42′14.86″ S 49°58′51.86″ W
C	2 ♂♀	Bacupari (RS), Brazil	30°28′41.01″ S 50°27′13.92″ W
D	2 ♀	Mostardas (RS), Brazil	31°06′17″ S 50°55′20″ W
E	2 ♂♀	Tavares (RS), Brazil	31°17′58.9″ S 51°05′47.6″ W
F	2 ♀	Bojuru (RS), Brazil	31°39′10.7″ S 51°26′14.8″ W
G	2 ♀	São José do Norte (RS), Brazil	32°04′34.47″ S 52°02′31.47″ W

SC = Santa Catarina and RS = Rio Grande do Sul, Brazilian States.

**Table 3:** Hybridization of eight microsatellites in seven cytotypes (A–G) of *Ctenomys minutus*.

Cytotypes	Motif Probes							
	(A)_30_	(C)_30_	(CA)_15_	(CAC)_10_	(CAG)_10_	(CGG)_10_	(GA)_15_	(GAG)_10_
Cyt A	Terminal and centromeric blocks on all chromosomes	Terminal blocks on all chromosomes and interstitial block on chr 1 and 8	Terminal and centromeric blocks on large chromosomes	Terminal blocks on all chromosomes and centromeric blocks on large chromosomes	Terminal and centromeric blocks on most chromosomes	Terminal blocks on large chromosomes and interstitial block on chr 8	Terminal blocks on all chromosomes	Terminal and centromeric blocks on all chromosomes
Cyt B	Terminal and centromeric blocks on all chromosomes	Terminal blocks on most chromosomes and interstitial block on chr 8	Terminal and centromeric blocks on all chromosomes	Terminal blocks on some chromosomes	Terminal blocks on some chromosomes	Terminal blocks on two chromosome pairs and interstitial block on chr 8	Terminal and interstitial blocks on all chromosomes	Terminal and interstitial blocks on some chromosomes
Cyt C	Terminal block on large chromosomes and centromeric block on all chromosomes	Terminal blocks on ten chromosome pairs and interstitial block on chr 1 and 8	Large blocks on all chromosomes	Terminal blocks on some chromosomes	Terminal and interstitial blocks on some chromosomes	Terminal blocks on some chromosomes and interstitial block on chr 8	Terminal and interstitial blocks on some chromosomes	Terminal and interstitial blocks on some chromosomes
Cyt D	Terminal and centromeric blocks on large chromosomes	Terminal blocks on all chromosomes and interstitial block on chr 8	Large blocks on larges chromosomes	Terminal and interstitial blocks on some chromosomes	Terminal and interstitial blocks on some chromosomes	Terminal blocks on ten chromosome pairs and interstitial block on chr 8	Terminal and interstitial blocks on all chromosomes	Terminal and interstitial blocks on all chromosomes
Cyt E	Terminal block on some chromosomes and centromeric block on some large chromosome	Terminal blocks on all chromosomes and interstitial block on chr 8	Large blocks in the q arm on all chromosomes	Terminal and proximal blocks on all chromosomes	Terminal and interstitial blocks on some chromosomes	Terminal blocks on four chromosome pairs and interstitial block on chr 8	Terminal and interstitial blocks on all chromosomes	Terminal and interstitial blocks on some chromosomes
Cyt F	Terminal block on some chromosomes and centromeric block on large chromosome	Terminal blocks on eight chromosome pairs and interstitial block on chr 1 and 8	Terminal blocks on some chromosomes	Terminal blocks on all chromosomes	Terminal blocks on some chromosomes	Interstitial block on chr 8	Terminal blocks on some chromosomes	Terminal blocks on some chromosomes
Cyt G	Terminal block on some chromosomes and centromeric block on some large chromosomes	Terminal blocks on six chromosome pairs and interstitial block on chr 8	Terminal and centromeric blocks on all chromosomes	Terminal and proximal blocks on all chromosomes	Terminal and proximal blocks on all chromosomes	Terminal blocks on four chromosome pairs and interstitial block on chr 8	Terminal and proximal blocks on all chromosomes	Terminal block on all chromosomes and centromeric blocks on chr 1

Chr = chromosome.

## Data Availability

Not applicable.

## Supplementary Materials

The following supporting information can be downloaded at: https://www.mdpi.com/article/10.3390/ani12162091/s1, Figure S1: Fluorescence in situ hybridization experiments with motif probes in cytotype A of *Ctenomys minutus*. Probes used are indicated in the lower right corner of the images and the cytotype in the lower left corner of the images. Figure S2: Fluorescence in situ hybridization experiments with motif probes in cytotype B of *Ctenomys minutus*. Probes used are indicated in the lower right corner of the images and the cytotype in the lower left corner of the images. Figure S3: Fluorescence in situ hybridization experiments with motif probes in cytotype C of *Ctenomys minutus*. Probes used are indicated in the lower right corner of the images and the cytotype in the lower left corner of the images. Figure S4: Fluorescence in situ hybridization experiments with motif probes in cytotype D of *Ctenomys minutus*. Probes used are indicated in the lower right corner of the images and the cytotype in the lower left corner of the images. Figure S5: Fluorescence in situ hybridization experiments with motif probes in cytotype E of *Ctenomys minutus*. Probes used are indicated in the lower right corner of the images and the cytotype in the lower left corner of the images. Figure S6: Fluorescence in situ hybridization experiments with motif probes in cytotype F of *Ctenomys minutus*. Probes used are indicated in the lower right corner of the images and the cytotype in the lower left corner of the images. Figure S7: Fluorescence in situ hybridization experiments with motif probes in cytotype G of *Ctenomys minutus*. Probes used are indicated in the lower right corner of the images and the cytotype in the lower left corner of the images.

## References

[1] López-Flores I., Garrido-Ramos M.A. (2012). The Repetitive DNA Content of Eukaryotic Genomes. Genome Dyn..

[2] Liehr T. (2021). Repetitive Elements in Humans. Int. J. Mol. Sci..

[3] Gibbs R.A., Weinstock G.M., Metzker M.L., Muzny D.M., Sodergren E.J., Scherer S., Scott G., Steffen D., Worley K.C., Burch P.E. (2004). Genome Sequence of the Brown Norway Rat Yields Insights into Mammalian Evolution. Nature.

[4] de Koning A.P.J., Gu W., Castoe T.A., Batzer M.A., Pollock D.D. (2011). Repetitive Elements May Comprise Over Two-Thirds of the Human Genome. PLoS Genet..

[5] Biet E., Sun J.-S., Dutreix M. (1999). Conserved Sequence Preference in DNA Binding among Recombination Proteins: An Effect of SsDNA Secondary Structure. Nucleic Acids Res..

[6] Liu Z., Li P., Kocabas A., Karsi A., Ju Z. (2001). Microsatellite-Containing Genes from the Channel Catfish Brain: Evidence of Trinucleotide Repeat Expansion in the Coding Region of Nucleotide Excision Repair Gene RAD23B. Biochem. Biophys. Res. Commun..

[7] Kidwell M.G. (2002). Transposable Elements and the Evolution of Genome Size in Eukaryotes. Genetica.

[8] Li Y., Korol A.B., Fahima T., Beiles A., Nevo E. (2002). Microsatellites: Genomic Distribution, Putative Functions and Mutational Mechanisms: A Review. Mol. Ecol..

[9] De Oliveira T.D., Kretschmer R., Bertocchi N.A., Degrandi T.M., De Oliveira E.H.C., De Cioffi M.B., Garnero A.D.V., Gunski R.J. (2017). Genomic Organization of Repetitive DNA in Woodpeckers (Aves, Piciformes): Implications for Karyotype and ZW Sex Chromosome Differentiation. PLoS ONE.

[10] Martins C., Pisano E., Ozouf-Costaz C., Foresti F., Kapoor B. (2007). Chromosomes and Repetitive DNAs: A Contribution to the Knowledge of the Fish Genome. Fish Cytogenetics.

[11] Komissarov A.S., Gavrilova E.V., Demin S.J., Ishov A.M., Podgornaya O.I. (2011). Tandemly Repeated DNA Families in the Mouse Genome. BMC Genom..

[12] Pokorná M., Kratochvíl L., Kejnovský E. (2011). Microsatellite Distribution on Sex Chromosomes at Different Stages of Heteromorphism and Heterochromatinization in Two Lizard Species (Squamata: Eublepharidae: *Coleonyx elegans* and Lacertidae: *Eremias velox*). BMC Genet..

[13] Cioffi M.d.B., Bertollo L.A.C. (2012). Chromosomal Distribution and Evolution of Repetitive DNAs in Fish. Repetitive DNA.

[14] Paço A., Adega F., Chaves R. (2014). *LINE-1* Retrotransposons: From ‘Parasite’ Sequences to Functional Elements. J. Appl. Genet..

[15] Bertocchi N.A., de Oliveira T.D., del Valle Garnero A., Coan R.L.B., Gunski R.J., Martins C., Torres F.P. (2018). Distribution of *CR1*-like Transposable Element in Woodpeckers (Aves Piciformes): Z Sex Chromosomes Can Act as a Refuge for Transposable Elements. Chromosom. Res..

[16] Wicker T., Sabot F., Hua-Van A., Bennetzen J.L., Capy P., Chalhoub B., Flavell A., Leroy P., Morgante M., Panaud O. (2007). A Unified Classification System for Eukaryotic Transposable Elements. Nat. Rev. Genet..

[17] Burton F.H., Loeb D.D., Voliva C.F., Martin S.L., Edgell M.H., Hutchison C.A. (1986). Conservation throughout Mammalia and Extensive Protein-Encoding Capacity of the Highly Repeated DNA Long Interspersed Sequence One. J. Mol. Biol..

[18] Boissinot S., Chevret P., Furano A.V. (2000). *L1* (*LINE-1*) Retrotransposon Evolution and Amplification in Recent Human History. Mol. Biol. Evol..

[19] Furano A., Duvernell D., Boissinot S. (2004). *L1* (*LINE-1*) Retrotransposon Diversity Differs Dramatically between Mammals and Fish. Trends Genet..

[20] Bratthauer G.L., Cardiff R.D., Fanning T.G. (1994). Expression of *LINE-1* Retrotransposons in Human Breast Cancer. Cancer.

[21] Esnault C., Maestre J., Heidmann T. (2000). Human *LINE* Retrotransposons Generate Processed Pseudogenes. Nat. Genet..

[22] Ovchinnikov I., Troxel A.B., Swergold G.D. (2001). Genomic Characterization of Recent Human *LINE-1* Insertions: Evidence Supporting Random Insertion. Genome Res..

[23] Acosta M.J., Marchal J.A., Fernández-Espartero C.H., Bullejos M., Sánchez A. (2008). Retroelements (*LINEs* and *SINEs*) in *Vole* Genomes: Differential Distribution in the Constitutive Heterochromatin. Chromosom. Res..

[24] Dobigny G., Ozouf-Costaz C., Waters P.D., Bonillo C., Coutanceau J.P., Volobouev V. (2005). *LINE-1* Amplification Accompanies Explosive Genome Repatterning in Rodents. Chromosom. Res..

[25] Appels R., Morris R., Gill B.S., May C.E. (1998). Chromosome Morphology and Number. Chromosome Biology.

[26] Teta P., D’Elía G. (2020). Uncovering the Species Diversity of Subterranean Rodents at the End of the World: Three New Species of Patagonian Tuco-Tucos (Rodentia, Hystricomorpha, *Ctenomys*). PeerJ.

[27] Reig O.A., Bush C., Ortells M.O., Contreras J.R., Nevo E., Reig O.A. (1990). An Overview of Evolution, Systematic, Population Biology, Cytogenetics, Molecular Biology and Speciation in *Ctenomys*. Evolution of Subterranean Mammals at the Organismal and Molecular Level.

[28] Freitas T.R.O., Wilson D., Lacer T., Mittermeier R. (2016). Family Ctenomyidae (Tuco-Tucos). Handbook of the Mammals of the World. Lagomorphs and Rodents I.

[29] Freitas T.R.O. (1997). De Chromosome Polymorphism in *Ctenomys minutus* (Rodentia-Octodontidae). Braz. J. Genet..

[30] Massarini A., Mizrahi D., Tiranti S., Toloza A., Luna F., Schleich C.E. (2002). Extensive Chromosomal Variation in *Ctenomys talarum talarum* from the Atlantic Coast of Buenos Aires Province, Argentina (Rodentia: Octodontidae). Mastozool. Neotrop..

[31] Freygang C.C., Marinho J.R., de Freitas T.R.O. (2004). New Karyotypes and Some Considerations about the Chromosomal Diversification of *Ctenomys minutus* (Rodentia: Ctenomyidae) on the Coastal Plain of the Brazilian State of Rio Grande Do Sul. Genetica.

[32] Freitas T.R.O. (2007). *Ctenomys lami*: The Highest Chromosome Variability in *Ctenomys* (Rodentia, Ctenomyidae) Due to a Centric Fusion/Fission and Pericentric Inversion System. Acta Theriol..

[33] Fernandes F., Fernández-Stolz G., Lopes C., Freitas T. (2007). The Conservation Status of the Tuco-Tucos, Genus *Ctenomys* (Rodentia: Ctenomyidae), in Southern Brazil. Braz. J. Biol..

[34] Freitas T.R.O. (1995). Geographic Distribution and Conservation of Four Species of the Genus Ctenomys in Southern Brazil. Stud. Neotrop. Fauna Environ..

[35] Gava A., de Freitas T.R.O. (2002). Characterization of a Hybrid Zone Between Chromosomally Divergent Populations of *Ctenomys minutus* (Rodentia: Ctenomyidae). J. Mammal..

[36] Gava A., Freitas T.R.O. (2004). De Microsatellite Analysis of a Hybrid Zone Between Chromosomally Divergent Populations of *Ctenomys minutus* from Southern Brazil (Rodentia: Ctenomyidae). J. Mammal..

[37] Lopes C.M., Ximenes S.S.F., Gava A., de Freitas T.R.O. (2013). The Role of Chromosomal Rearrangements and Geographical Barriers in the Divergence of Lineages in a South American Subterranean Rodent (Rodentia: Ctenomyidae: *Ctenomys minutus*). Heredity.

[38] Freitas T.R.O. (2006). De Cytogenetics Status of Four *Ctenomys* Species in the South of Brazil. Genetica.

[39] Gava A., Freitas T.R.O. (2003). Inter and Intra-Specific Hybridization in Tuco-Tucos (*Ctenomys*) from Brazilian Coastal Plains (Rodentia: Ctenomyidae). Genetica.

[40] Fornel R., Cordeiro-Estrela P., de Freitas T.R.O. (2018). Skull Shape and Size Variation within and between *mendocinus* and *torquatus* Groups in the Genus *Ctenomys* (Rodentia: Ctenomyidae) in Chromosomal Polymorphism Context. Genet. Mol. Biol..

[41] Ditcharoen S., Bertollo L.A.C., Ráb P., Hnátková E., Molina W.F., Liehr T., Tanomtong A., Triantaphyllidis C., Ozouf-Costaz C., Tongnunui S. (2019). Genomic Organization of Repetitive DNA Elements and Extensive Karyotype Diversity of Silurid Catfishes (Teleostei: Siluriformes): A Comparative Cytogenetic Approach. Int. J. Mol. Sci..

[42] Sember A., De Oliveira E.A., Ráb P., Bertollo L.A.C., De Freitas N.L., Viana P.F., Yano C.F., Hatanaka T., Marinho M.M.F., De Moraes R.L.R. (2020). Centric Fusions behind the Karyotype Evolution of Neotropical *Nannostomus pencilfishes* (Characiforme, Lebiasinidae): First Insights from a Molecular Cytogenetic Perspective. Genes.

[43] Rossi M.S., Pesce C.G., Reig O.A., Kornblihtt A.R., Zorzópulos J. (1993). Retroviral-like Features in the Monomer of the Major Satellite DNA from the South American Rodents of the Genus *Ctenomys*. Mitochondrial DNA.

[44] Novello A., Cortinas M.N., Suárez M., Musto H. (1996). Cytogenetic and Molecular Analysis of the Satellite DNA of the Genus Ctenomys (Rodentia Octodontidae) from Uruguay. Chromosom. Res..

[45] Sikes R.S. (2016). The animal Care and use committee of the american Society of mammalogists 2016 Guidelines of the American Society of Mammalogists for the Use of Wild Mammals in Research and Education. J. Mammal..

[46] Verma R., Babu A. (1996). Human Chromosomes: Principles & Techniques. Mol. Reprod. Dev..

[47] Kubat Z., Hobza R., Vyskot B., Kejnovsky E. (2008). Microsatellite Accumulation on the Y Chromosome in *Silene latifolia*. Genome.

[48] Doyle J.J., Doyle J.L. (1987). A Rapid DNA Isolation Procedure for Small Quantities of Fresh Leaf Tissue. Phytochem. Bull..

[49] Casavant N.C., Scott L., Cantrell M.A., Wiggins L.E., Baker R.J., Wichman H.A. (2000). The End of the *LINE*? Lack of Recent *L1* Activity in a Group of South American Rodents. Genetics.

[50] Richard G.-F., Kerrest A., Dujon B. (2008). Comparative Genomics and Molecular Dynamics of DNA Repeats in Eukaryotes. Microbiol. Mol. Biol. Rev..

[51] Liehr T., Kosayakova N., Schröder J., Ziegler M., Kreskowski K., Pohle B., Bhatt S., Theuss L., Wilhelm K., Weise A. (2011). Evidence for Correlation of Fragile Sites and Chromosomal Breakpoints in Carriers of Constitutional Balanced Chromosomal Rearrangements. Balk. J. Med. Genet..

[52] Ruiz-Herrera A., Castresana J., Robinson T.J. (2006). Is Mammalian Chromosomal Evolution Driven by Regions of Genome Fragility?. Genome Biol..

[53] Ferguson-Smith M.A., Trifonov V. (2007). Mammalian Karyotype Evolution. Nat. Rev. Genet..

[54] Fan Y., Newman T., Linardopoulou E., Trask B.J. (2002). Gene Content and Function of the Ancestral Chromosome Fusion Site in Human Chromosome 2q13-2q14.1 and Paralogous Regions. Genome Res..

[55] Kehrer-Sawatzki H., Schreiner B., Tänzer S., Platzer M., Müller S., Hameister H. (2002). Molecular Characterization of the Pericentric Inversion That Causes Differences between Chimpanzee Chromosome 19 and Human Chromosome 17. Am. J. Hum. Genet..

[56] Locke D.P., Archidiacono N., Misceo D., Cardone M.F., Deschamps S., Roe B., Rocchi M., Eichler E.E. (2003). Refinement of a Chimpanzee Pericentric Inversion Breakpoint to a Segmental Duplication Cluster. Genome Biol..

[57] Kehrer-Sawatzki H., Sandig C.A., Goidts V., Hameister H. (2005). Breakpoint Analysis of the Pericentric Inversion between Chimpanzee Chromosome 10 and the Homologous Chromosome 12 in Humans. Cytogenet. Genome Res..

[58] Leffak M. (2017). Break-Induced Replication Links Microsatellite Expansion to Complex Genome Rearrangements. BioEssays.

[59] Balzano E., Pelliccia F., Giunta S. (2021). Genome (in)Stability at Tandem Repeats. Semin. Cell Dev. Biol..

[60] Gadgil R.Y., Romer E.J., Goodman C.C., Dean Rider S., Damewood F.J., Barthelemy J.R., Shin-Ya K., Hanenberg H., Leffak M. (2020). Replication Stress at Microsatellites Causes DNA Double-Strand Breaks and Break-Induced Replication. J. Biol. Chem..

[61] Feng W., Di Rienzi S.C., Raghuraman M.K., Brewer B.J. (2011). Replication Stress-Induced Chromosome Breakage Is Correlated with Replication Fork Progression and Is Preceded by Single-Stranded DNA Formation. G3 Genes Genomes Genet..

[62] Glover T.W. (2006). Common Fragile Sites. Cancer Lett..

[63] Schwartz M., Zlotorynski E., Kerem B. (2006). The Molecular Basis of Common and Rare Fragile Sites. Cancer Lett..

[64] Yakovchuk P., Protozanova E., Frank-kamenetskii M.D. (2006). Base-Stacking and Base-Pairing Contributions into Thermal Stability of the DNA Double Helix. Nucleic Acids Res..

[65] Volff J., Körting C., Meyer A., Schartl M. (2001). Evolution and Discontinuous Distribution of *Rex3* Retrotransposons in Fish. Mol. Biol. Evol..

[66] Ferreira D.C., Porto-Foresti F., Oliveira C., Foresti F. (2011). Transposable Elements as a Potential Source for Understanding the Fish Genome. Mob. Genet. Elements.

[67] Araújo N.P., Sena R.S., Bonvicino C.R., Kuhn G.C.S., Svartman M. (2021). *SINE-B1* Distribution and Chromosome Rearrangements in the South American *Proechimys gr. goeldii* (Echimyidae, Rodentia). Cytogenet. Genome Res..

[68] Vieira-da-Silva A., Adega F., Guedes-Pinto H., Chaves R. (2016). *LINE-1* Distribution in Six Rodent Genomes Follow a Species-Specific Pattern. J. Genet..

[69] Slamovits C.H., Cook J.A., Lessa E.P., Susana Rossi M. (2001). Recurrent Amplifications and Deletions of Satellite DNA Accompanied Chromosomal Diversification in South American Tuco-Tucos (Genus *Ctenomys*, Rodentia: Octodontidae): A Phylogenetic Approach. Mol. Biol. Evol..

[70] Erickson I.K., Cantrell M.A., Scott L., Wichman H.A. (2011). Retrofitting the Genome: L1 Extinction Follows Endogenous Retroviral Expansion in a Group of Muroid Rodents. J. Virol..

[71] Feschotte C., Pritham E.J. (2006). Mobile DNA: Genomes under the Influence. Genome Biol..

[72] Waminal N.E., Pellerin R.J., Kang S.H., Kim H.H. (2021). Chromosomal Mapping of Tandem Repeats Revealed Massive Chromosomal Rearrangements and Insights Into *Senna tora* Dysploidy. Front. Plant Sci..

