# Supplementary material for: Genomic Organization of Microsatellites and LINE-1-like Retrotransposons: Evolutionary Implications for Ctenomys minutus (Rodentia: Ctenomyidae) Cytotypes
Source: Animals (Basel). 2022 Aug 16;12(16):2091. doi: 10.3390/ani12162091 (PMC9405301; doi:10.3390/ani12162091)
Supplement: Supplementary file 1 [file animals-12-02091-s001.zip › animals-1786317-supplementary.pdf]

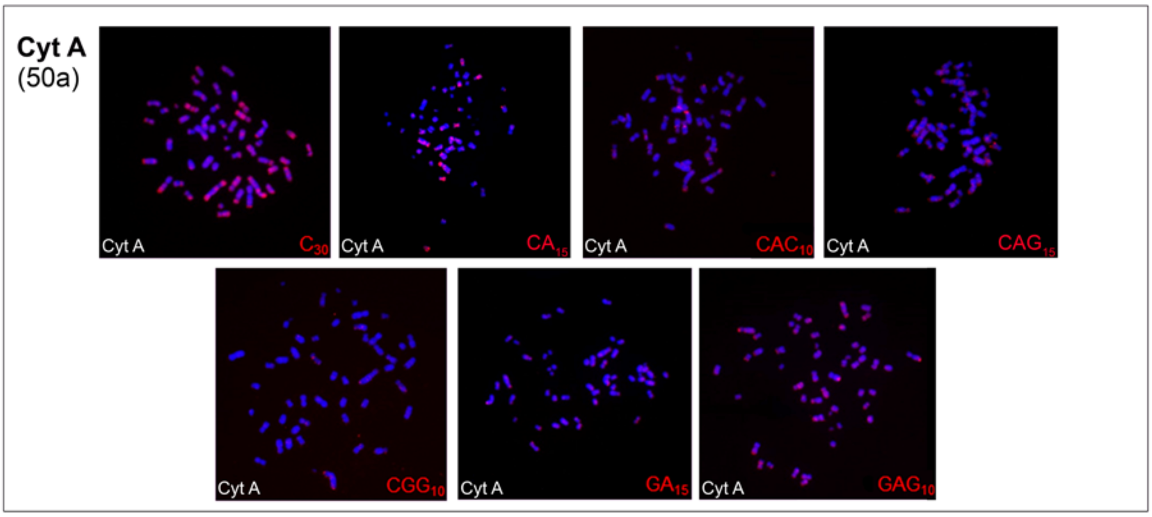

Figure S1. Fluorescence in situ hybridization experiments with motif probes in cytotype A of *Ctenomys minutus*. Probes used are indicated in the lower right corner of the images and the cytotype in the lower left corner of the images.

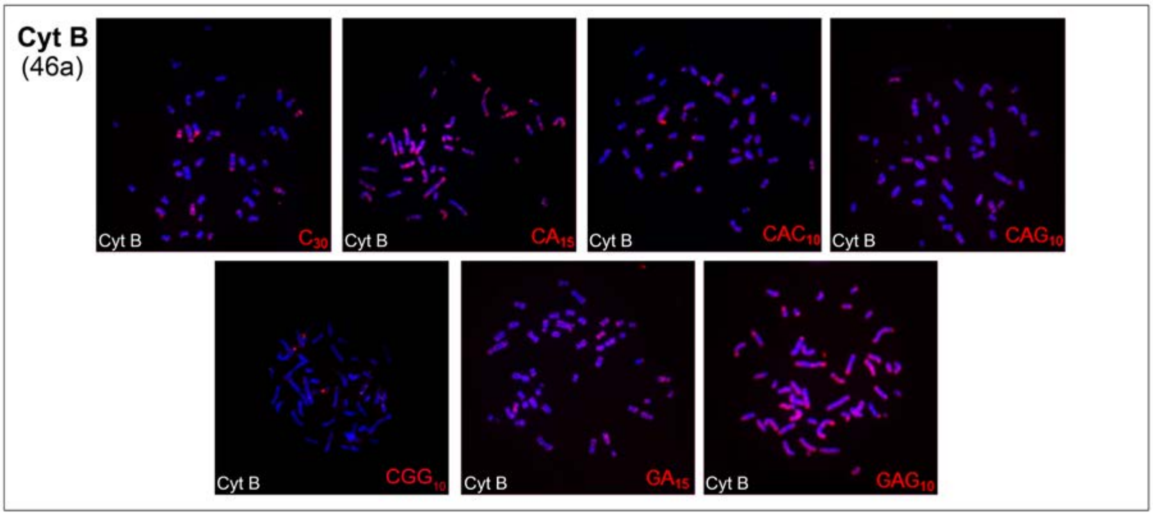

Figure S2. Fluorescence in situ hybridization experiments with motif probes in cytotype B of *Ctenomys minutus*. Probes used are indicated in the lower right corner of the images and the cytotype in the lower left corner of the images.

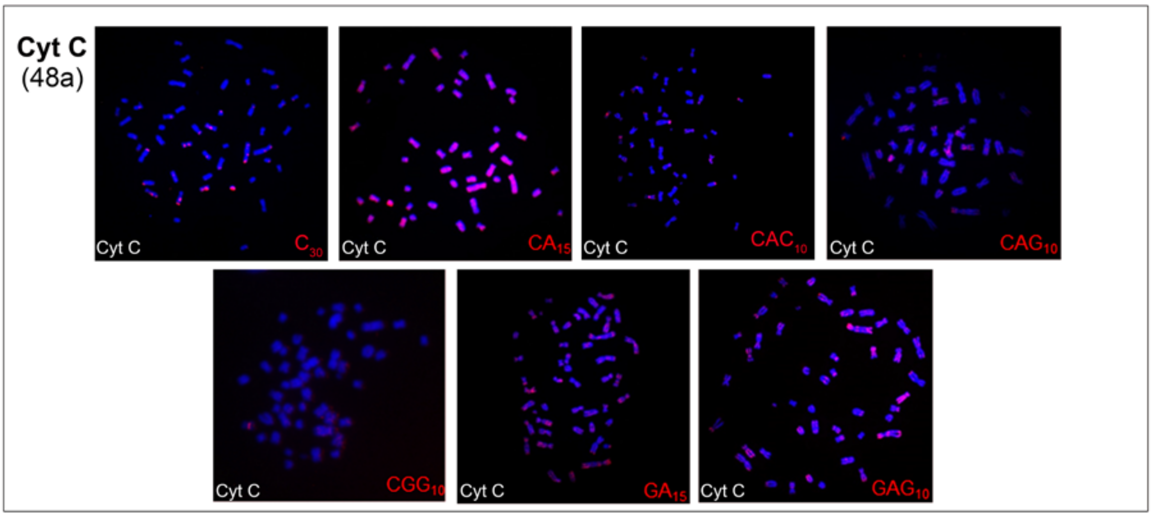

Figure S3. Fluorescence in situ hybridization experiments with motif probes in cytotype C of *Ctenomys minutus*. Probes used are indicated in the lower right corner of the images and the cytotype in the lower left corner of the images.

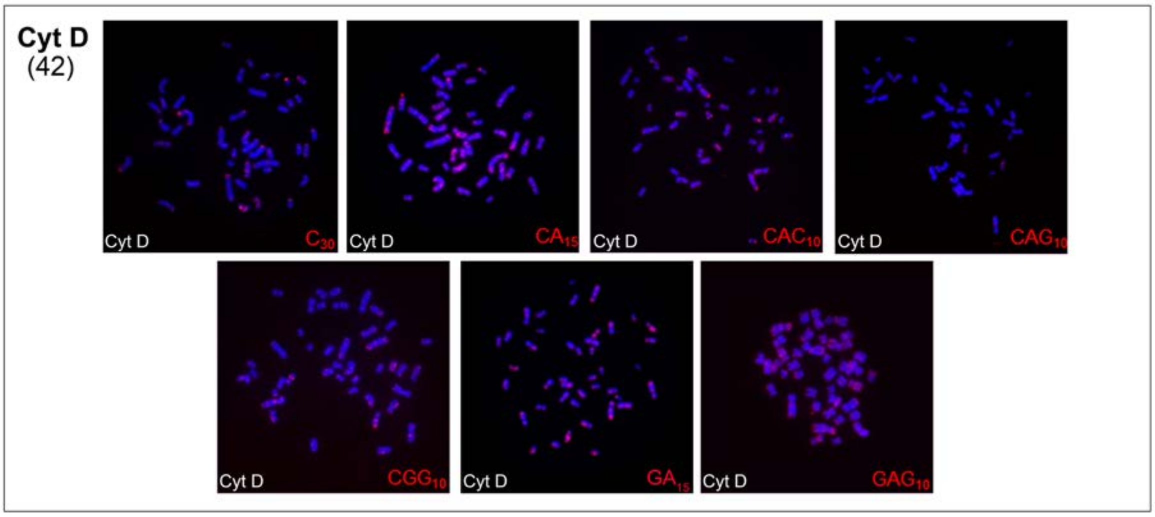

Figure S4. Fluorescence in situ hybridization experiments with motif probes in cytotype D of *Ctenomys minutus*. Probes used are indicated in the lower right corner of the images and the cytotype in the lower left corner of the images.

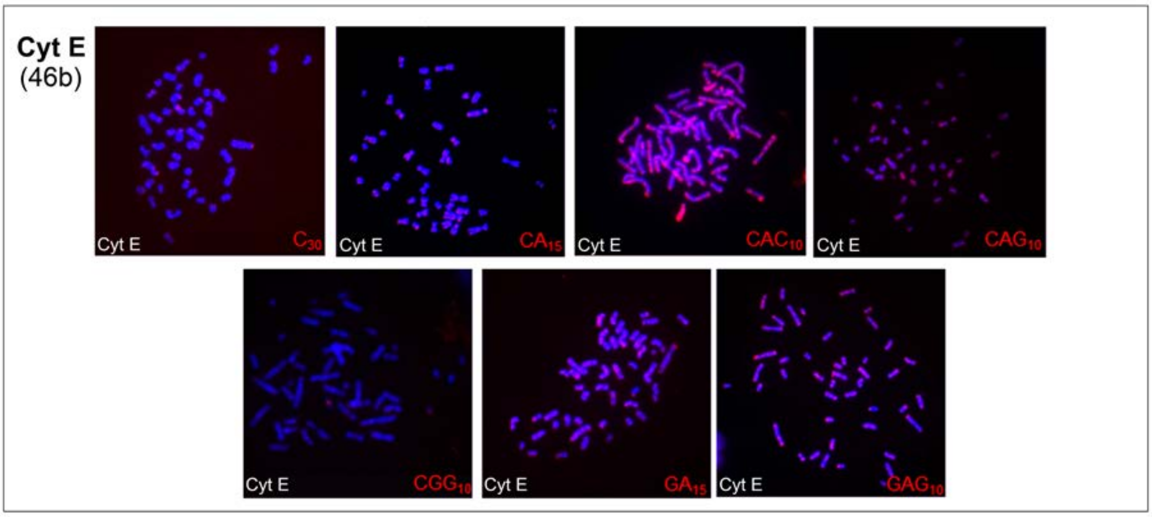

Figure S5. Fluorescence in situ hybridization experiments with motif probes in cytotype E of *Ctenomys minutus*. Probes used are indicated in the lower right corner of the images and the cytotype in the lower left corner of the images.

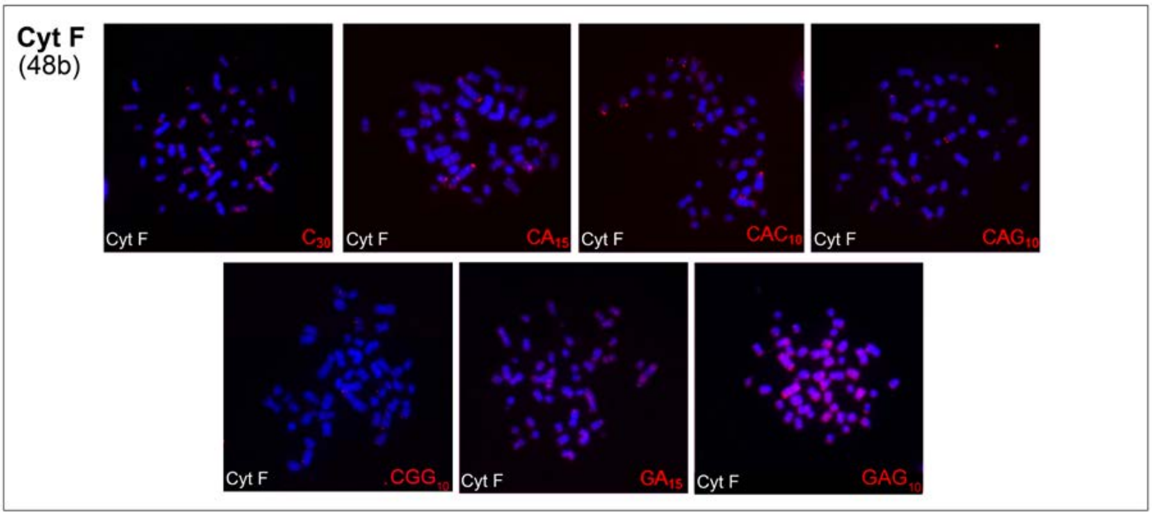

Figure S6. Fluorescence in situ hybridization experiments with motif probes in cytotype F of *Ctenomys minutus*. Probes used are indicated in the lower right corner of the images and the cytotype in the lower left corner of the images.

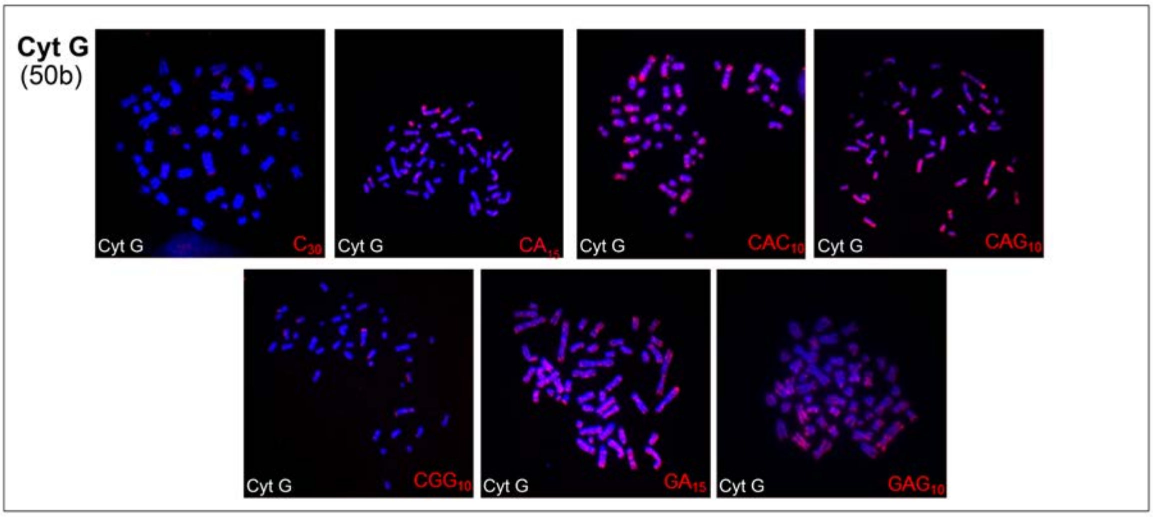

Figure S7. Fluorescence in situ hybridization experiments with motif probes in cytotype G of *Ctenomys minutus*. Probes used are indicated in the lower right corner of the images and the cytotype in the lower left corner of the images.
